# Comparison of canine colostrum and milk using a multi-omics approach

**DOI:** 10.1186/s42523-024-00309-4

**Published:** 2024-04-22

**Authors:** Alisa Cohen, Sondra Turjeman, Rachel Levin, Smadar Tal, Omry Koren

**Affiliations:** 1 Azrieli Faculty of Medicine, Bar-Ilan University, Safed, Israel; 2 Koret School of Veterinary Medicine, The Hebrew University Veterinary Teaching Hospital, Hebrew University of Jerusalem, Rehovot, Israel; 3 Tel-Hai Academic College, Upper Galilee, Israel; 4 Kyung Hee University, Seoul, the Republic of Korea

**Keywords:** Canine, Colostrum, Microbiota dynamics, Milk, Untargeted metabolomics

## Abstract

**Background:**

A mother’s milk is considered the gold standard of nutrition in neonates and is a source of cytokines, immunoglobulins, growth factors, and other important components, yet little is known about the components of canine milk, specifically colostrum, and the knowledge related to its microbial and metabolic profiles is particularly underwhelming. In this study, we characterized canine colostrum and milk microbiota and metabolome for several breeds of dogs and examined profile shifts as milk matures in the first 8 days post-whelping.

**Results:**

Through untargeted metabolomics, we identified 63 named metabolites that were significantly differentially abundant between days 1 and 8 of lactation. Surprisingly, the microbial compositions of the colostrum and milk, characterized using 16S rRNA gene sequencing, were largely similar, with only two differentiating genera. The shifts observed, mainly increases in several sugars and amino sugars over time and shifts in amino acid metabolites, align with shifts observed in human milk samples and track with puppy development.

**Conclusion:**

Like human milk, canine milk composition is dynamic, and shifts are well correlated with developing puppies’ needs. Such a study of the metabolic profile of canine milk, and its relation to the microbial community, provides insights into the changing needs of the neonate, as well as the ideal nutrition profile for optimal functionality. This information will add to the existing knowledge base of canine milk composition with the prospect of creating a quality, tailored milk substitute or supplement for puppies.

**Supplementary Information:**

The online version contains supplementary material available at 10.1186/s42523-024-00309-4.

## Background

A mother’s milk is considered the ideal form of nourishment for mammalian newborns [1–3]. It is composed of lipids, proteins, carbohydrates and micronutrients necessary for neonatal growth [2, 4–6]. In addition, it is a source of cytokines, immunoglobulins, growth factors, soluble receptors, immune cells, enzymes, and commensal microbes [7]. Colostrum, the milk produced by the mammary glands immediately after parturition, plays several key roles in neonatal development, including passive transfer of maternal antibodies and antimicrobial agents. It also influences early microbial colonization of offspring, and supplies energy for the newborn [8–12], and later milk continues to shape puppies’ immune profiles and microbiota maturation trajectories.

Little is known about the components of canine milk, specifically colostrum, and the knowledge related to its microbial and metabolic profiles is particularly underwhelming [13]. Following whelping, a puppy’s gut is seeded with microbes from various sources, one of which is the dam’s milk [14, 15]. Milk microbes are associated with a range of functionalities including amino acid and lactose metabolism and genes that code for cellular respiration, signaling, antibiotic resistance, and stress-related functions [5, 16, 17]. Metabolites obtained directly from the milk and from bacteria transferred from the milk are also diverse [18, 19] and support the myriad functional benefits of suckling and breast feeding. The human breast milk microbiome and metabolome have been widely studied and are known to play crucial roles in providing immunity and shaping the formation of the neonatal immune system [20–23]. Deep milk profiling is still lacking in canines though; to our knowledge, no untargeted metabolite profiling has been performed. Although canine milk metabolome reports in the literature do exist, they are focused on specific classes of metabolites and do not consider profiles in the context of the milk microbiome. One study found marked differences in canine milk compared to that of bovine and caprine milk with the former having higher levels of proteins and unsaturated fats, as well as a number of minerals, but lower levels of saturated fats and lactose [24]. Another study specifically characterized oligosaccharide profiles, which were determined to be sensitive to sample collection time and host diet [4].

In this study, we characterized canine colostrum and milk microbiota and metabolome of 24 dams (of 11 breeds of dogs) and examined profile shifts as milk matures in the first 8 days post-whelping. Such a study of the metabolic profile of canine milk, and its relation to the microbial community, could provide insights into the needs of the neonate, as well as the ideal profile for optimal functionality. This information will add to the existing knowledge base of canine milk composition with the prospect of creating a high-quality, tailored substitute or supplement for puppies.

## Results

### Dam characteristics

This study includes milk samples from 24 dams collected at two time points, the first within 24 h of parturition, considred colostrum (day 1), and the second, one week later (day 8; Table 1), referred to as milk. In total, five kennels/breeding locations and 11 dog breeds were sampled. The median age of dams in the study was four (range: 2-6.5 years), and the most sampled breed was the Border Collie.

**Table 1:** Demographics of the dams included in this study

Birth no.	Samples Processed		Breed	Kennel	Age (years)	Live pups	Food brand
	Colostrum	Milk					
2	Mic, Met	Mic, Met	Poodle	B	2	4	Royal Canin
4	Mic, Met	Mic, Met	Border Collie	M	5.5	8	Bil Jac
10	Mic, Met	Mic, Met	Border Collie	M	2.5	6	Bil Jac
11	Met	Met	Shetland Sheepdog	N	4	3	Novopet
13	Mic, Met	Mic, Met	Border Collie	N	3.5	8	Monge
14	Mic, Met	Mic, Met	Jack Russell	N	5	5	Monge
15	Mic, Met	Mic, Met	Cavalier King Charles Spaniel	S	6	3	Mixed*
16	Mic, Met	Mic, Met	German Shepherd	S	4	5	Mixed*
17	Mic, Met	Mic, Met	Cavalier King Charles Spaniel	S	6	4	Mixed*
18	Mic, Met	Met	Shih Tzu	S	5	4	Mixed*
19	Mic, Met	Mic, Met	Australian Shepherd	N	6	8	Monge
21	Met	Met	Labrador	G	4	9	Pro Plan Salmon
22	Mic, Met	Met	Border Collie	M	4	4	NA
23	Met	Mic, Met	Australian Shepherd	N	2.5	5	NA
24	Met	Met	Australian Shepherd	N	6	6	NA
25	Met	Mic, Met	Cavalier King Charles Spaniel	S	4	3	Mixed*
26	Met	Met	Border Collie	N	5	7	Royal Canin Starter
27	Met	Mic, Met	Sarplaninac	N	5	7	Royal Canin Starter
28	Mic, Met	Mic, Met	Australian Kelpie	N	4	8	NA
29	Met	Met	Labrador	G	3.7	7	Pro Plan Salmon
30	Mic, Met	Mic, Met	Border Collie	N	4	10	NA
31	Mic, Met	Mic, Met	Border Collie	M	5	4	Monge
32	Met	Mic, Met	Border Collie	N	3.5	6	Royal Canin
33	Mic, Met	Mic, Met	Border Collie	N	6.5	2	Royal Canin

Twenty-four dams were sampled within 24 h of whelping and 8 days later to obtain colostrum and milk samples. Data including sampling location (Kennel), the number of live puppies birthed in sampled litter (Live pups), and primary diet were recorded, and the types of samples processed at each timepoint for each dam are also specified (Mic: microbiome sample processed and passed quality control; Met: untargeted metabolomics sample processed). NA: not available; B: hobby breeder 1; G: Israel Guide Dog Center for the Blind; M: Mishmar HaEmek Kennel; N: Roey Hakfar Kennel; S: Dog’s Life Kennel. *Mixed diet: 50% Pro Plan performance, 50% equal parts: Royal Canin Starter, Pro Plan Medium Salmon Puppies, Natural Balance for Small Dogs

### Microbiome

**Fig. 1:**
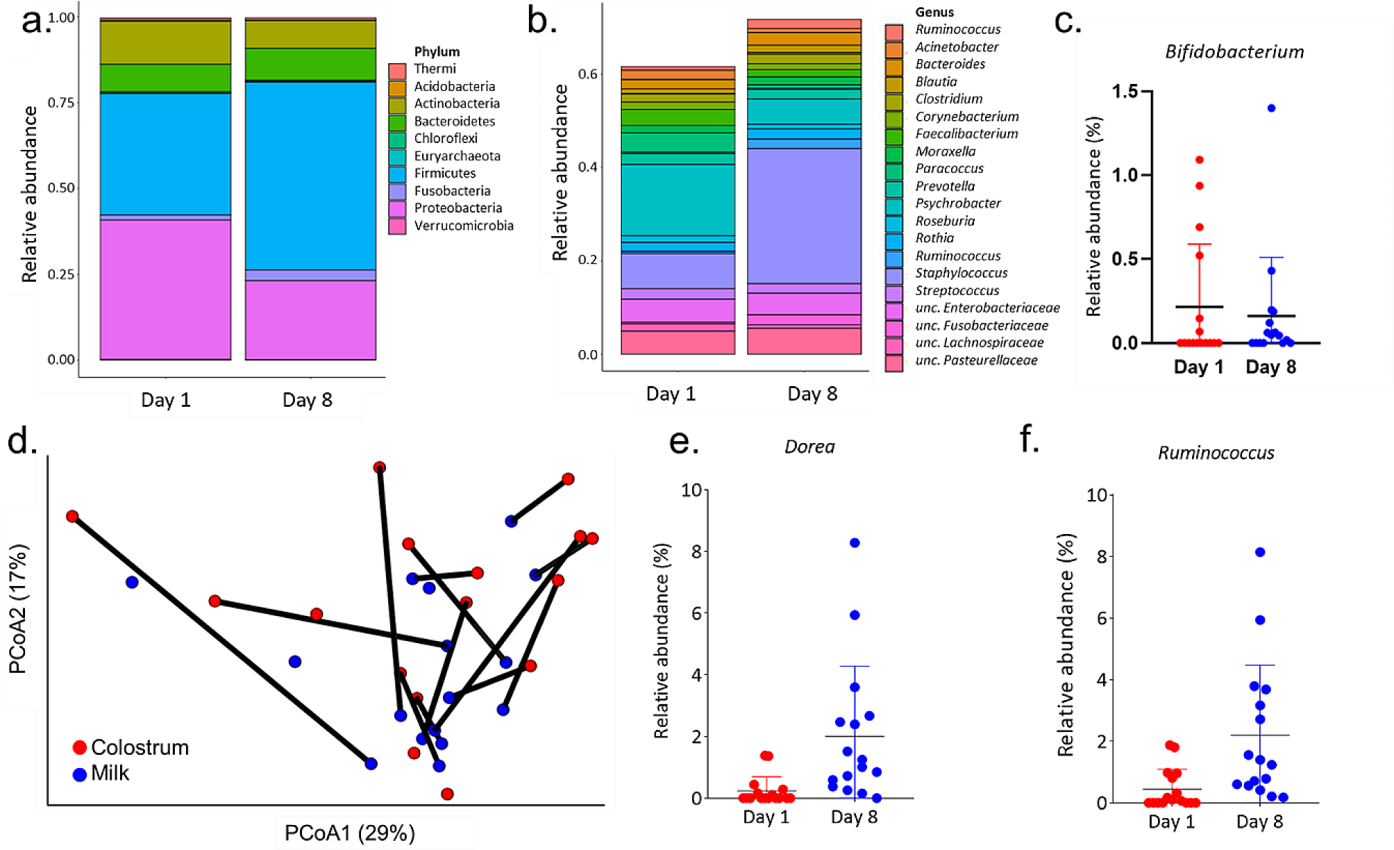
Microbiota of canine milk on days 1 and 8 post-whelping. Colostrum and milk samples were processed for 16S rRNA gene sequencing, and the microbiota were characterized. Relative abundance at the (**a**) phylum level (10 most abundant taxa) and (**b**) genus level (20 most abundant taxa), after rarefaction, are displayed divided by colostrum samples (day 1) and milk samples (day 8). Remainders to 1.00 are the fraction of the other less abundant taxa. (**c**) Relative abundance of the genus *Bifidobacterium*, a highly abundant taxon in human milk was examined. While prevalence was 37.5% and 62.5% for colostrum and milk, respectively, as seen here, abundance was low and did not change with time post-whelping. (**d**) Principal coordinate analysis representing beta diversity based on weighted UniFrac distances of dams at two time points: day 1 (red), and day 8 (blue) post-whelping. Lines connecting the dots show the two timepoints of a single dam, while isolated dots represent samples for which only one timepoint passed quality control (PERMANOVA, *p*-value = 0.133). Differential abundance analysis revealed increased relative abundance of (**e**) *Dorea* (ANCOM, W = 382) and (**f**) *Ruminococcus* (ANCOM, W = 357) in milk samples compared to colostrum. N_colostrum_=16, N_milk_=16

The milk microbiome was characterized with amplicon sequencing of the V4 region of the 16S rRNA gene [25]. Of the 48 samples collected (24 dams, 2 timepoints each), 32 were successfully processed and passed quality control (16 colostrum and 16 milk samples: 13 paired samples and 6 additional, unpaired samples; Table 1). Bacterial composition of canine milk was then characterized at each timepoint. The phyla Firmicutes, Proteobacteria, Actinobacteria and Bacteroidetes were the most abundant in the samples overall, and Fusobacteria were more abundant in milk than colostrum (Fig. 1a, ANCOM (analysis of compositions of microbiomes) W = 5). The genera *Staphylococcus* and *Psychrobacter*, as well as unclassified members of the families Enterobacteriaceae and Pasteurellaceae, dominated canine colostrum and milk (Fig. 1b). While it was not among the dominant genera of the canine milk microbiota, it is worth noting that the genus *Bifidobacterium*, a genus prevalent in human milk, was also present in both colostrum (37.5% of samples) and milk (62.5%), though at very low abundances (mean < 0.25%) (Fig. 1c).

When comparing the composition of the microbiota at the two timepoints (weighted UniFrac visualized with principal coordinate analysis), colostrum samples appeared to be more scattered, while the milk sampled on day 8 were more closely clustered (comparison of within-group sample to sample distances, t-test, *p*-value < 0.001). There was, however, no significant difference in the compositions across days (PERMANOVA (permutational multivariate analysis of variance), *p*-value = 0.133, Fig. 1d). Similarly, no significant differences in alpha diversity (measured by Faith’s phylogenetic distance (PD) metric) between the two timepoints were observed (Mann-Whitney, *p*-value = 0.598). Differential abundance analysis (ANCOM) revealed that the genera *Dorea* and *Ruminococcus* were significantly more abundant in milk than in colostrum (*Dorea*, W = 382, *Ruminococcus*, W = 357, Fig. 1e, f).

### Metabolome

**Fig. 2:**
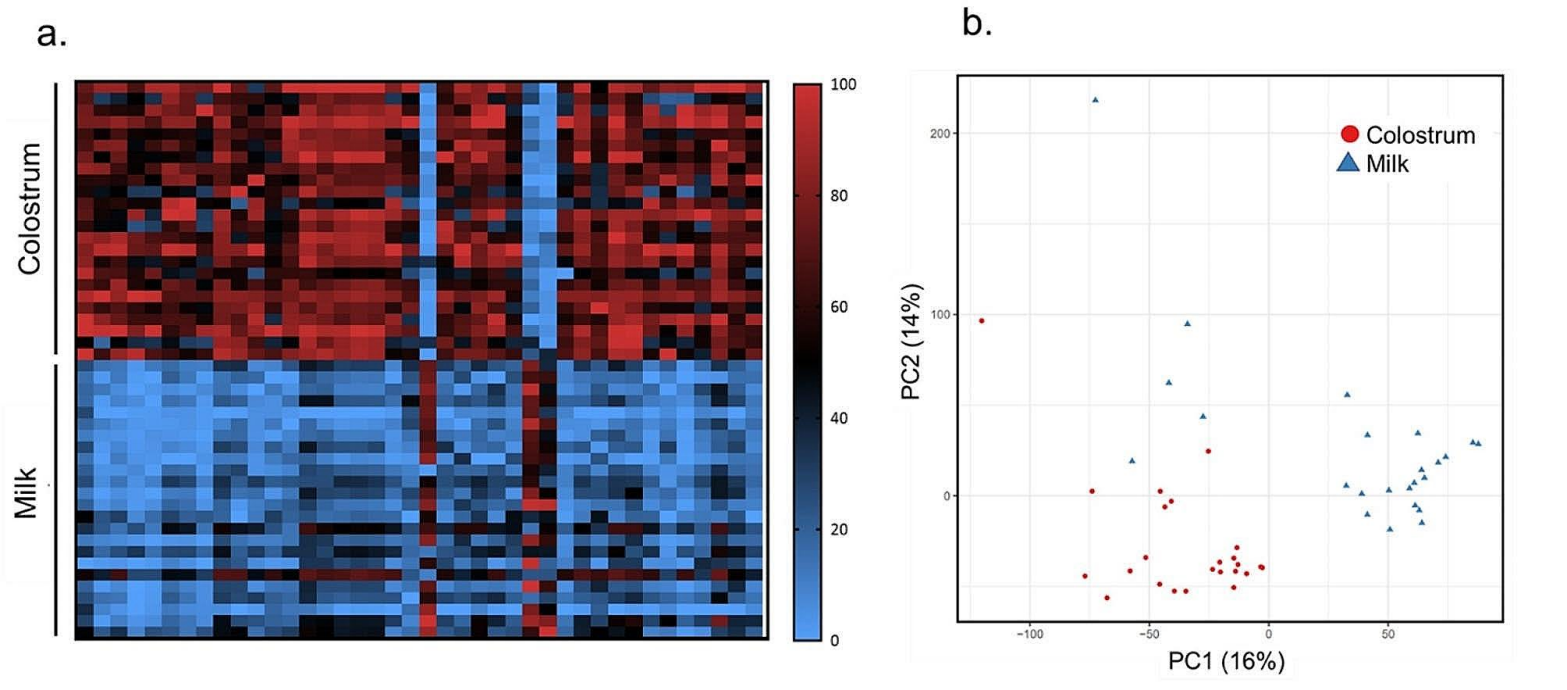
Milk metabolome compared across two timepoints. LC-MS was used for untargeted metabolomics of canine colostrum (*N* = 24) and milk (*N* = 24) collected eight days apart. (**a**) A heatmap of metabolites separated by time of lactation, normalized to range from 0-100%. Red signifies upregulated metabolites and blue signifies downregulated metabolites. (**b**) Principal component analysis (PCA) of the overall metabolite profile, showing the first two components that explain 16% and 14% of the variability between samples, respectively. When coloring samples by timepoint, a clear separation is observed signifying different colostrum and milk metabolome profiles

Untargeted metabolomics (liquid chromotography-mass spectromtry, LC-MS) was used to characterize the colostrum and milk metabolomes and then profiles were compared. Of the metabolites detected, only a subset could be named against relevant databases (see Methods). Three-hundred ninety metabolites were significantly differentially abundant between the two timepoints (q-value < 0.05, Fig. 2a), of which, less than 70 could be named (Table S1). Visual examination of the principal component analysis (PCA) plot shows a clear separation in metabolome profiles based on the time of lactation (Fig. 2b). Among the differentially expressed metabolites were several sugars, including lactulose, galactose, N-Acetylneuraminic acid, pantothenic acid 4'-O-b-D-glucoside, and glycan 6’-sialyllactose increased after 8 days of lactation.

### Microbiome-metabolome interactions

**Fig. 3:**
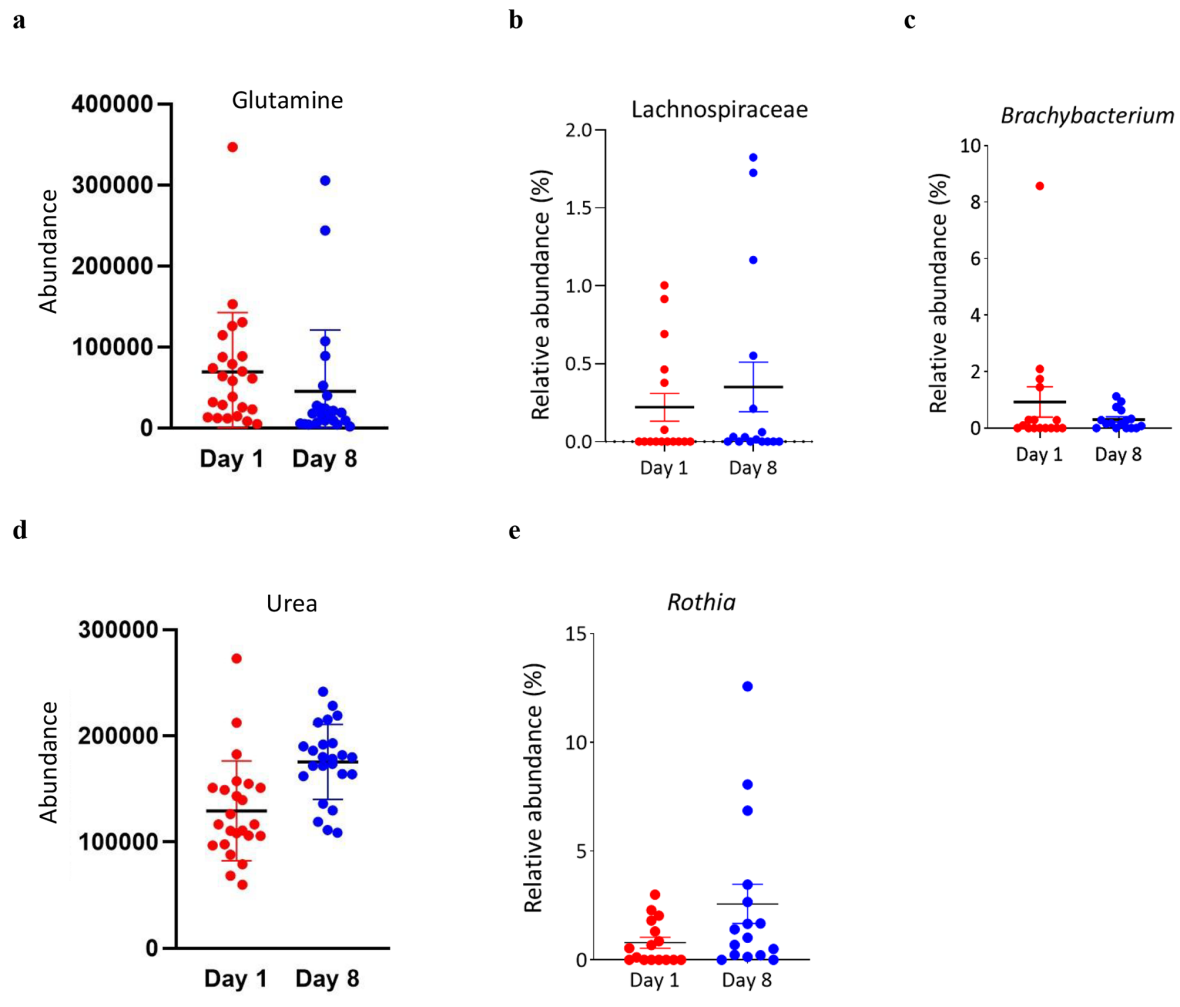
Microbe-metabolite correlations found by MIMOSA2. Following profiling of the colostrum and milk microbiota (*N*_*colostrum*_*=16, N*_*milk*_*=16*) and metabolome (*N*_*colostrum*_*=24, N*_*milk*_*=24*), we used MIMOSA2 to identify differentially abundant microbially-associated metabolites across timepoints. (**a**) L-glutamine abundance (paired Welch’s t-test, FDR-adjusted *p*-value = 0.0310) was associated with the bacterial taxa (**b**) Lachnospiraceae (inversely related with L-glutamine; t-test: *p*-value = 0.4789) and (**c**) *Brachybacterium* (positively related with L-glutamine; t-test: *p*-value = 0.2658). (**d**) Urea abundance (paired Welch’s t-test, FDR-adjusted *p*-value = 0.0003) was positively related with (**e**) *Rothia* abundance (t-test, *p*-value = 0.0651)

Integration of microbiota and metabolome profiles using Model-based Integration of Metabolite Observations and Species Abundances (MIMOSA2) [26], revealed that the amino acid L-glutamine decreased after 8 days of lactation, mainly due to changes in the Lachnospiraceae family and the genus *Brachybacterium* (Fig. 3a-c). Conversely, urea increased during lactation progression and correlated with changes in the genus *Rothia* (Fig. 3d-e). No other metabolites of microbial origin were observed to differ significantly between the colostrum and milk samples.

## Discussion

The current study investigated both the microbial and metabolite profiles of canine milk in the immediate postpartum period (colostrum) and one week later. Although multiple named metabolites were significantly differentially expressed between days 1 and 8 of lactation, the microbial compositions of the colostrum and milk were, surprisingly, largely similar, with only two differentiating genera. This could suggest that the milk microbiome is relatively stable and that the functional profiles for newborn and week-old puppies’ needs may be similar. Although differences between the timepoints were not found, a notable pattern of within-group beta-diversity was observed, with colostrum samples being significantly more divergent and day 8 milk samples converging across dams. In addition, increased abundance of members of the Fusobacteria phylum was observed in canine milk compared to colostrum, a pattern not generally observed in longitudinal studies of human milk [27, 28], though a genus of the Fusobacteria phylum (*Leptotrichia*) was observed to be more abundant in 1- and 6-month postpartum milk samples than in human colostrum [28]. The findings of significantly different metabolite profiles in the colostrum and milk could suggest that while the bacterial players are largely the same, their roles shift, leading to differential production of metabolites as milk matures or that non-bacterial dynamics underlie these changes. Inclusion of bacterial meta-transcriptional analyses in the future can help determine if and how the microbiota behave differently in the total community at these two unique timepoints. Alternatively, it is possible that in some cases, milk rather than colostrum was sampled in the 24 h post-whelping window, or that the naturally low microbial biomass of milk samples could result in a low diversity of reads [29].

### Milk microbiome characterization

*Staphylococcus* was the most abundant genus in canine milk in the current study (%, mean ± s.d.: colostrum: 6.4 ± 7.6, milk: 24.4 ± 19.4) and has been reported by Boix-Amorós et al. [30] as one of the core genera present in human breast milk, along with *Streptococcus* (here: 2.4 ± 6.0 and 2.2 ± 2.4%, respectively) and *Corynebacterium* (here: 1.7 ± 2.3 and 1.3 ± 2.5%, respectively). *Staphylococcus* and *Streptococcus* are known to utilize oxygen and are thought to be ideal pioneer colonizers of the neonate gut as they can prepare the environment for beneficial anaerobic commensals [31]. *Acinetobacter*, another one of the core genera found in human breast milk [30], was also abundant in the canine milk (%, colostrum: 1.1 ± 2.8, milk: 0.7 ± 1.1), although not among the 10 most abundant genera, and its specific role in the milk/neonate microbiome is yet unknown [32, 33]. Interestingly, while the genera *Finegoldia*, *Peptoniphilus* and *Pseudomonas* were detected in canine milk, they were found in very low abundance (mean < 0.1%), unlike their dominant nature in human breast milk [30]. In bovine milk, only *Pseudomonas* is common [32, 33]. The genus *Psychrobacter* dominated both canine colostrum (17.7 ± 26.3%) and milk (6.7 ± 14.4%). While this genus has been reported in both human and cow’s milk [34, 35], it was far from being as predominant in those species as it is in canines. To our knowledge, while *Psychrobacter* has been reported in canine skin [36], stool [37] and meconium [14], it has not been identified as the dominating genus in canine milk until now. This genus is thought to have a role in the breakdown of organic carbons other than sugars [38]. Interestingly, in human milk, *Bifidobacterium* is a central bacterial player, known to metabolize human milk oligosaccharides. It is a key component in the human milk microbiota, which can modulate newborn immune development [39]. In cow’s milk, *Bifidobacterium* species are also present and able to degrade bovine milk oligosaccharides [40]. In our canine study, while observed in both colostrum and milk, *Bifidobacterium* prevalence (37.5 and 62.5% respectively) and abundance (0.2 ± 0.3 and 0.1 ± 0.3%, respectively) were very low. Taken together, this suggests that while some bacterial taxa are conserved across species, the milk microbiota in canines also consists of uniquely abundant features. Both the genera *Ruminococcus* and *Dorea*, members of the Lachnospiraceae family, were significantly more abundant in milk than colostrum. The Lachnospiraceae family is known for its ability to produce short chain fatty acides (SCFAs) [41], and there is evidence of increased SCFAs as human milk matures [42], suggesting synchrony between the microbiome, milk SCFA profile, and the infant’s nutritional needs.

### Milk metabolome characterization

Among the various components of breast milk, human milk oligosaccharides are complex sugars that serve as metabolic substrates for gut microbiota with antimicrobial activity [43]. Within this class of molecules, free oligosaccharides in breast milk represent an interesting and dynamic component and have been known to vary by species and time of lactation [44]. In a previous study, 3’-sialyllactose, 6’-sialyllactose, and 2’-fucosyllactose accounted for over 90% of all oligosaccharides in canine milk [4]. In our study, 2’-fucosyllactose was not detected, but 3’-sialyllactose and 6’-sialyllactose were prominent. In this study, several sugars, such as lactulose, a natural laxative and sugar known to be a prebiotic for important bacterial taxa like *Bifidobacterium* [45], galactose, N-acetylneuraminic acid, pantothenic acid 4'-O-b-D-glucoside, and glycan 6’-sialyllactose, increased after 8 days of lactation. Interestingly, 3’-sialyllactose decreased. Consistent with these results, a study that identified milk oligosaccharides in canines observed a decrease of 3’-sialyllactose levels in the first 10 days of lactation. This oligosaccharide is thought to be associated with altricial growth [44], which could explain its decrease eight days post-whelping. We also observed a significant increase in amino sugars, such as N-acetylglucosamine, glycan 6’-sialyllactose amine 6-phosphate, and N-acetyl-glucosamine 6-phosphate, after 8 days of lactation. In humans, amino sugars serve as a key byproduct of human milk oligosaccharide metabolism and are components of bacterial cell walls [46]. The increased levels of amino sugars during lactation could be in line with the natural progression of microbial colonization and maturation of suckling puppies [43].

We next investigated the differences in amino acid metabolites in canine milk. We found a significant decrease in key amino acids including arginine, histidine, lysine, and phenylalanine during lactation. Similar observations of reduced total and essential amino acids have been reported in human breast milk in two systematic reviews [47, 48], and it is suggested that while the nutritional value of milk is relatively consistent, the developing neonate is more dependent on the high protein content of colostrum than older infants [47]. Other amino acid derivatives, including alanine betaine, histidine betaine, glycine betaine, urea, and prolinamide, increased with time from whelping, while trimethyl lysine decreased during lactation. There is evidence of increases in free fatty acids, in human milk as well, as time since birth increases [48].

Previous studies have shown that nucleotides and nucleosides are important bioactive compounds in milk with significant regulatory factors in different mammalian species [49]. These compounds are known to play an essential role in energy production, metabolism, and signaling [50]. We identified several nucleosides, nucleotides, and their analogues that were differentially abundant in the colostrum and milk samples. Notably, the levels of adenosine, methylguanine, UDP-N-acetylglucosamine, and xanthine were increased after 8 days of lactation. Similar observations of increased nucleotide and nucleoside levels have been previously reported in canine milk [50]. This increase was attributed to an increase in food intake and *de novo* synthesis. Deoxycytidine, methyl-adenosine, and N6 − threonylcarbamoyl-adenosine decreased after 8 days of lactation. The lower deoxycytidine levels might be explained by the compound’s role as a cofactor in phospholipid biosynthesis [51]. Due to the biochemical properties of nucleosides and nucleotides, the European Commission allows for their supplementation to human baby formula [49], and there are already similar canine supplements (e.g. Nucleoforce Dogs [52]) and nucleotide-enriched chows (e.g. Arden Grange [53]). This practice might also benefit newborn puppies that are fed commercial puppy formulas.

Changes in complex lipids were identified during lactation, including for glycerophospholipids and sphingomyelin (Table S1). In humans and other animals, these lipids have been known to play crucial roles in the physiological function and stability maintenance of milk fat globule membrane [54, 55]. They are even involved in proper brain development and neuron differentiation in humans; presence of similar lipids in canines could suggest shared functionality across the animal kingdom [56].

Carnitine is an essential nutrient, and it plays a key role in fatty acid metabolism and cellular energy production [57, 58]. Carnitine binds to fatty acids and generates various acylcarnitines which transport activated long-chain fatty acids into the mitochondria for β-oxidation as a major source of energy for cellular activities [59]. It was reported that in cow’s milk, the concentration of acylcarnitines decreases during the first 2 months of lactation. However, in humans, the levels remain unchanged [60]. Here, we observed that the levels of L-carnitine and deoxycarnitine increased as lactation progressed while the levels of acetyl carnitine, acylcarnitine 16:1, hexanoylcarnitine, iso-valeryl carnitine, octanoyl carnitine, oleoyl carnitine, and propionyl carnitine decreased, more consistent with bovine milk dynamics than human ones.

Energy metabolites such as succinic acid and creatine were decreased after 8 days of lactation. In addition to its involvement in ATP generation in the mitochondria, succinic acid acts as an inflammatory signal molecule. Moreover, increased circulating succinic acid levels have been linked to impaired glucose metabolism and specific modifications in the gut bacteria in obese individuals [61]. Accordingly, succinic acid may be beneficial in colostrum to induce rapid neonate growth but could then decrease in abundance among healthy mothers as offspring growth stabilizes, as was observed here.

### Microbiome-metabolome interactions

When examining the relationship between the microbiota and metabolite profiles, we identified a significant association between two metabolites and several microbial counterparts. First, two microbial taxa were found to be responsible for the decrease of L-glutamine in canine milk: the genus *Brachybacterium* and the Lachnospiraceae family. Several strains of the genus *Brachybacterium* have previously been isolated from milk products [62, 63] and cow’s milk [34]; however, their role in milk digestion and newborn wellbeing is still poorly understood. Taxa from the Lachnospiraceae family have also been reported in cow’s milk [64]. L-glutamine, or glutamine, is a conditionally essential amino acid, and it is one of the most abundant amino acids in human breast milk [48]; it is also prevalent in the milk of other mammals [65, 66]. Glutamine plays an important role in neonatal growth and development [67]. High glutamine content in milk is most likely related to the high glutamine needs of the neonate, promoting rapid growth and cell division, particularly in the neonatal small intestine [68]. Here we found increased abundance of the metabolite in colostrum suggesting that in newborn puppies, the need is even greater than 8-day-old ones. It has been proposed by Manso et al. (2012) that dietary glutamine supplementation can improve the growth and intestinal health of piglets throughout the suckling period [68]. Plaizier et al. (2001) performed glutamine infusions in cows and found that the plasma concentrations of glutamine increased significantly [69]. Glutamine supplementation of dams immediately post-whelping might also be beneficial to canines. In the current study, we found that members of the genus *Brachybacterium* were positively correlated with glutamine. Perhaps supplementation with *Brachybacterium* probiotics could serve to increase glutamine levels in milk.

In addition, we found a positive correlation between the genus *Rothia* and the metabolite urea. Urea is an abundant metabolite in breast milk [70] and in the milk of other mammals [34, 65, 71], and urea is among the metabolites that have been reported to be conserved in breast milk due to its important roles in infant growth and development [72–74]. The connection between *Rothia* and urea is of interest as *Rothia* was previously reported in breast milk [35], and *Rothia dentocariosa*, specifically, has been reported to be a urease-positive strain, meaning that it is a bacterial species in which urease is frequently isolated [75]. While we can’t be sure which strain of the *Rothia* genus was found in the canine milk, the correlation between this genus and urea suggests that it might also be urease-positive. Typically *Bifidobacterium* is associated with urea in human milk as it is known for its ability to use urea as a main source of nitrogen [70]. In canine milk, *Bifidobacterium* abundance was low, but *Rothia* was positively correlated with urea, suggesting that *Rothia* in canines may play a role parallel to that of *Bifidobacterium* in humans.

### Study limitations

One of the main limitations of this study was the small sample size. While inclusion of 24 individuals is in line with other microbiota-metabolome studies, the variance of our sample was not evenly disributed with most breeds being represented by only one individual and one being represented by 9. Similarly, in several cases, effects of breed, diet and/or kennel may not be independent. Future studies should have a more balanced design and increased sample size– or focus on only a single breed and kennel, in parallel to many mouse studies– to fully understand the various variables at play. Another limitation of the study stems from the nature of available databases, for naming both bacteria and metabolites, and their suitability for non-model (or even non-human) organisms. Many metabolites are unnamed and only a few microbially-derived metabolite differences were observed. As the field progresses, the data generated in this study can be reanylzed to deepen our understanding of milk maturation dynamics. While many may see the use of 16S rRNA sequencing as a limitation, to date, whole genome sequencing methods are still largely unreliable for milk as the microbial DNA content is much lower than the human DNA content in the samples.

## Conclusions

This study provides an integrative characterization of canine colostrum and milk, extensively profiling the microbiome and metabolome. Many bacterial taxa and metabolites characterized in canine milk have also been observed in bovine and human milk offering support for a core milk functional profile maintained, at least in part, by the microbiome. Future work examining dam-puppy microbiota dynamics and health status can shed further light on how the milk microbiome and metabolome affect developing puppies.

### Methods

#### Study design and sample collection

The study included purebred dogs from four breeding kennels and one hobby breeder located throughout northern and central Israel (1–12 dams per kennel, median = 4). Eleven different breeds were sampled, with 1–9 dams sampled per breed (median = 1). Because most breeds were represented by only a single dam, a single kennel, and a single diet, we could not include breed as an explanatory or random variable in our analyses.

Kennel managers notified us when dams whelped, and then we visited dams on days one (within 24 h) and eight postpartum. Milk was milked directly into sterile centrifuge tubes via manual stimulation of the mammary glands and nipples and immediately frozen in a portable freezer. The samples were also transferred in the portable freezer at -20 °C and then stored in a -80 °C freezer until processing. Milk collected within 24 h of whelping was considered colostrum and milk collected on day eight was referred to as milk. Dam demographics, litter characteristics, and the brand of dog food were recorded. All procedures were approved by the Hebrew University’s Ein Karem animal ethics committee (Approval # MD-21-16495-2).

### Microbiome characterization

Bacterial DNA from milk samples was extracted using the MasterPure Complete DNA & RNA Purification kit (Epicentre, Madison, WI) according to the manufacturer’s instructions, following centrifugation for the removal of the fat fraction, a subsequent two-minute bead beating step, and enzyme incubation with lysozyme for improved degradation. Extracted DNA was amplified in a two-step, nested PCR reaction with 343F-806R primers which target the V3-V4 region of the 16S rRNA gene [76] (5 cycles), followed by an amplification with 515F-806R primers which target the V4 region of the 16S rRNA gene (30 cycles, using barcoded primers) [25]. Following PCRs, samples were purified (Kapa Pure Beads, Roche, Basel, Switzerland), quantified (Quant-iT™ PicoGreen™ dsDNA Assay, Thermo Fisher Scientific, Waltham, MA), and pooled in equimolar amounts. All samples were sequenced on the Illumina MiSeq platform (Genomic Center, Azrieli Faculty of Medicine, Bar-Ilan University, Israel) using the 300 cycle MiSeq V2 reagent kit (Illumina, San Diego, CA). Sequencing data were preprocessed and analyzed with QIIME2 [77], version 2021.11 and amplicon errors were corrected using the DADA2 pipeline [78]. Single end sequences were grouped by feature, and taxonomy was assigned using the GreenGenes database [79].

### Untargeted metabolomics

Untargeted liquid chromatography–mass spectrometry (LC-MS) based metabolic profiling was performed at Afekta Technologies Ltd. (Kuopio, Finland) using reversed-phase (RP) and hydrophilic interaction chromatography (HILIC), with positive and negative electrospray ionization (ESI). A total of 48 canine milk samples from 24 dams at two time points (day 1 and day 8 of lactation) were analyzed. The samples used for this analysis were duplicates of those used for microbiome characterization, for the purpose of integrating the two analyses (Table 1).

The samples were thawed over ice, vortexed and centrifuged at 4500 × g and + 4 °C for 10 min. The available supernatant volume (10, 25, 40, 50, 80, or 100 µL) was collected and moved to a separate tube. Cold aqueous methanol (80%) was added to the supernatant, and each sample was immediately vortexed for 30 s after the addition of methanol. The samples were centrifuged at 12,000 × g and + 4 °C for 15 min. Finally, the samples were filtered into vials at room temperature using syringe filters (PALL Acrodisc 13 mm with 0.2 μm PTFE membrane) and a pooled quality control (QC) sample was collected from all the samples, with the exception of samples where the amount of supernatant was only 10 µL.

Liquid chromatography–mass spectrometry analysis was performed on an Agilent 6546 Q-TOF LC-MS System with Agilent Jet Stream source and a 1290 Infinity II Ultra-High-Performance Liquid Chromatography (UHPLC) system. A Zorbax Eclipse XDB-C18 column (2.1 × 100 mm, 1.8 μm; Agilent Technologies) was used for the RP separation and an Aqcuity ultra-performance liquid chromatography (UPLC) BEH amide column (Waters) was used for the HILIC separation. After each chromatographic run, the ionization was carried out using jet stream ESI in the positive and negative mode, yielding four data files per sample. The collision energies for the MS/MS analysis were 10, 20, and 40 V, for compatibility with spectral databases.

Peak detection and alignment were performed in MS-DIAL ver. 4.90 [80]. For the peak collection, m/z values between 50 and 1500 and all retention times were considered. The amplitude of minimum peak height was set at 3000, and they were detected using the linear weighted moving average algorithm. For the alignment of the peaks across samples, the retention time tolerance was 0.1 min and the m/z tolerance was 0.015 Da. The solvent background was removed using solvent blanks such that the signal abundance across the samples had to be at least five times that of the average in the blanks.

After the peak picking, a total of 64,802 molecular features were included in the data preprocessing and clean-up step. Low-quality features were flagged and discarded from statistical analyses according to the following quality metrics: low number of missing values (present in more than 70% of the QC samples, present in at least 50% of samples in at least one study group), RSD* (relative standard deviation) below 20%, and D-ratio* (dispersion ratio) below 10%. In addition, if either RSD* or D-ratio* was above the threshold, the features were kept if their classic RSD, RSD* and basic D-ratio were all below 10%. The signals were normalized for signal drift. Missing values were imputed using Random Forest imputation for high-quality features or simple imputation with value of 0 for low-quality features. After the preprocessing and data clean-up, 17,454 molecular features were considered of good quality and included in the FDR (false discovery rate) correction calculations (see statistical analyses, below). Metabolite naming was done using an internal library of 1,000 compounds and the MassBank [81], MoNA (Mass Bank of North America, https://mona.fiehnlab.ucdavis.edu/) databases, as well as others available from the RIKEN Center for Sustainable Resource Science website. Data were processed and analyzed using R [82].

### Statistical analysis

Differences in microbial alpha diversity (within-sample diversity or richness) were assessed using Faith’s phylogenetic diversity (Faith’s PD) [83] and compared with Mann Whitney tests due to the non-normal nature of the residuals. The weighted UniFrac method was applied to evaluate beta diversity (between-sample diversity) [84] and differences in the microbiota at the two timepoints were assessed by PERMANOVA. ANCOM was used to identify differentially abundant microbial features between the groups [85] using default parameters in the QIIME2 plugin. Following metabolite normalization (described above), paired Welch’s t-tests with FDR corrections for multiple comparisons were used to compare metabolite abundances across the two time points. The model-based integration of metabolite observations and species abundances version 2 (MIMOSA2) tool was used to integrate the microbiome and metabolomics data [26].

### Electronic supplementary material

Below is the link to the electronic supplementary material.

**Table S1**: Identified significantly differential compounds by milk type. Identified significantly differential compounds with class information and and statistical data. Cohen’s D signifies the effect size: a positive value means higher average abundance the milk versus colostrum. The raw *p*-values and Benjamini-Hochberg false discovery rate (FDR) corrected q-values are shown (attached as a separate.xlsx).

## Data Availability

Sequencing data has been uploaded and can be found at EBI under the accession number ERP157004. The metabolomic data is available in the online supplement.
